# Supplementary material for: Are carbon emissions trading and green financial instruments synergistic? -Comprehensive quantitative research based on content analysis
Source: PLoS One. 2024 Mar 7;19(3):e0298601. doi: 10.1371/journal.pone.0298601 (PMC10919601; doi:10.1371/journal.pone.0298601)
Supplement: S1 Data — (ZIP) [file pone.0298601.s001.zip › Data/地级市Z_F工作报告.zip!/╔╜╬≈/┬└┴║╩╨/└┤╫╘╣1⁄2╓┌║┼í╛┬φ┐╦╩2╛▌═°í┐_2017.docx]

　　现在，我代表市人民政府向大会报告工作，请予审议，并请市政协委员和其他列席人员提出意见。

　　一、2016年工作回顾

　　2016年，我们深入学习贯彻党的十八大和十八届三中、四中、五中、六中全会精神及习近平总书记系列重要讲话精神，按照省委“一个指引、两手硬”重大思路和要求，在市委的坚强领导下，团结带领全市广大干部群众提振信心、顽强拼搏，奋力推动全市经济企稳回升、稳中向好，较好地完成了全年主要任务，实现了省委提出的“下半年好于上半年，为2017年进一步好转奠定坚实基础”的目标。

　　全年地区生产总值完成995.3亿元，同比增长4.1%，增速排名从2015年的全省末位上升到第6位；规模以上工业增加值完成546.7亿元，增长2.6%，从2015年全省末位上升到第4位；一般公共预算收入完成89.6亿元，下降1.2%，从2015年全省第10位上升到第5位；社会消费品零售总额完成433.7亿元，增长6.8%。固定资产投资完成1118.5亿元，下降4.1%。城镇居民人均可支配收入24180元，增长5.6%；农村居民人均可支配收入7644元，增长6.3%。万元 GDP 能耗、二氧化碳排放量和用水量降幅，二氧化硫、粉尘、烟尘等减排幅度，均按要求完成了省定任务。市区优良天气数达到287天，环境空气质量综合指数全省排名第2。一年来，主要抓了以下工作:

　　——以脱贫攻坚统揽经济社会发展全局，脱贫攻坚首战首胜。举全市之力打出“3105”政策组合拳，全面开展“三个一”扶贫行动，精准实施“十项重点工程”，扎实推进“五项机制”创新。按照“五统一两集中”模式，推进光伏扶贫迈出坚实步伐，方山建成全省最大的村级集中式光伏电站。“吕梁山护工”品牌开始叫响省城、京城。经济林提质增效进展顺利。易地扶贫搬迁全省现场会在我市召开，投资完成率和总竣工率分别超省26和11个百分点。“8+2”农业产业化继续推进，生态脱贫探索形成“合作社+贫困户”“政府购买式造林”等模式，得到国务院领导的高度肯定。建成219处农村饮水安全工程和14个水质检测中心，10万农村人口饮水标准得到提升。出台医疗救助“16条”和教育扶贫系列措施，城乡低保和五保供养标准大幅提高，兜底工作逐步夯实。加强第一书记队伍建设,推进了县际结对帮扶工作。278个贫困村、11.1万人脱贫,脱贫攻坚实现首战告捷。

　　——抢抓机遇推动转型升级，产业结构初现新格局。主动走出去与全国排名前十的河南明泰、嵩基、杭州鼎胜等铝精深加工企业达成合作意向，推动铝产业向下游延伸。兴华科技2×35万吨铝基新材料、华兴铝业二期100万吨氧化铝项目投产，全省最大的轻铝合金生产线中铝华润一期50万吨项目开工建设。积极盘活企业闲置资源资产，大力引进高科技、金融资本和优秀人才，加快煤焦产业纵向延伸，推动交城华鑫与太原理工大学、孝义鹏飞与北京三聚环保、孝义金岩与山东科瑞合作，鹏飞集团建成全国领先的甲醇联产 LNG 项目。聘请著名导演贾樟柯对我市旅游开发进行策划、包装。7月份在太原举办了旅游推介暨项目招商大会，推出了一批旅游产品和项目。7月份华为公司大数据中心项目落地离石，10月份北京软通动力城市创新综合体项目落地孝义，中交网通智慧高速大数据中心项目落户交城。全市煤电铝、煤焦化、大数据等产业集群化、集聚化发展格局初步形成。

　　——坚定不移抓好供给侧结构性改革，发展新动能持续增强。严格落实“三去一降一补”，煤炭行业核减产能1060万吨，水泥行业退出落后产能45万吨。坚持与棚户区改造、移民搬迁、采煤沉陷区治理相结合，制定化解房地产库存、购房补贴等政策，减少库存商品房172.4万平米。积极防范金融风险，联盛集团破产重整依法有序推进；农信社改制步伐加快，孝义农商行挂牌运营；引导企业通过合资合作、债转股等方式，处置银行不良贷款23.7亿元。全市1328名干部深入439个重点企业开展帮扶服务；组织300名民营企业家、1000名企业经营管理人员在浙江大学等院校培训，民营企业创新转型意识和发展信心明显增强。坚决落实省市税费减免政策，减少企业税负4亿元，减征社会保险费1亿元。推动17户用电企业与发电企业直接交易电量23.1亿度，节约电费1.16亿元。市区公交运营体制改革基本完成，100辆新能源公交车投入运营。积极推广运用 PPP 项目融资模式，分三批次向社会发布总投资1138亿元的105个项目，在建 PPP 项目达19个。组织“迎老乡、邀客商、兴吕梁”、北京和汾阳农产品展销推介等系列招商活动，香港王老吉、天津宝迪、北京新瑞利邦等一批行业龙头企业落户吕梁。

　　——狠抓基础设施建设，城乡面貌明显改善。集中精力开展新区安置和市区重点项目建设“百日大会战”，新区16个安置区69栋楼主体工程及配套设施完工，供热、供水、供电、供气、通讯、雨污等市政管线建设基本完成，1000户征地拆迁群众拿到新房钥匙。城建“双十”重点工程加快推进，市医疗卫生园区、儿童福利院开工建设，市图书馆、群艺馆启动前期工作，吕梁大道一期绿化、主城区人行天桥、便民公厕等一批工程完工。积极化解市游泳馆遗留问题，完成维修改造。祁离、静兴2条高速和3条国省道改线前期工作进展顺利。成立市交通投资平台，承接农发行贷款40亿元，新建和改造农村公路769公里。吕梁至太原开通了城际列车，极大地方便了群众出行。

　　——持续加大民生投入，人民福祉进一步增强。全市民生支出226.7亿元，占公共预算支出的82.2%。优先发展教育事业，新建、改扩建农村幼儿园26所，文水、交口、中阳等6县通过国家义务教育均衡发展评估认定。吕梁学院附中投入使用。整合离石师范、吕梁教育学院等学校资源，筹建吕梁高等师范专科学校，招生1140名。加快发展医疗卫生事业，我市被确定为第二批国家级医养结合试点市，120个农村老年人日间照料中心建成。1.15万名贫困残疾人得到康复救助。积极组织参加省政府《人说山西好风光》电视竞演活动，全市人民广泛参与、积极支持，扩大了吕梁对外影响力。城镇新增就业3.2万人，失业率控制到2.72%。完成营造林57.34万亩、吕梁山生态脆弱区造林47.03万亩，完成水土流失治理43万亩、生态修复22万亩。开展“铁腕治污”行动，实施冬季城乡环境集中整治。毫不松懈抓安全，全市未发生重大以上事故，安全生产形势总体平稳。深入开展以打黑除恶为重点的“三大战役”，刑事案件和“两抢一盗”发案数大幅下降，有力维护了社会大局稳定。

　　——切实加强政府自身建设，施政能力和服务水平有效提升。深入开展“两学一做”学习教育，落实全面从严治党主体责任，持续加强政府系统党风廉政建设和反腐败工作。积极完善议事决策程序，政府依法行政能力进一步增强。严格执行省政府“13710”工作制度，政府工作人员的执行力和落实力明显提高。认真办理人大代表建议和政协委员提案，办结率达到100％。支持各人民团体工作，国防动员、人民防空、民兵预备役建设进一步加强；粮食供销、外事侨务、文物保护、防震减灾、民族宗教、档案方志、应急管理、对台、气象等工作取得新的进步。

　　各位代表，回顾过去的一年，面对经济持续下行的困难局面，我们毫不动摇地贯彻党的基本路线，坚持以经济建设为中心，把全面构建良好政治生态最终体现到调动广大党员干部、人民群众和企业家的积极性、推动经济发展上来，干部群众精气神得到提升，企业家投资创业的信心逐渐增强，地区生产总值结束了2014年以来连续9个季度的下滑态势，规模以上工业增加值扭转了连续30个月的下行局面，一般公共预算收入降幅逐步收窄，一些标志性、转折性、趋势性变化增加，经济发展已经走出下滑下行、低速低位的最困难时期。一年来，我们认真贯彻新发展理念，把发展的主攻方向放在改革开放、转型升级上，坚持走出去、请进来，到北京，到上海、浙江等地考察学习、招商引资，聘请麦肯锡公司制定全市产业发展规划，铝工业、煤焦化、大数据等产业集群化发展取得突破性进展，局域电网建设为主的电力体制改革、华为和军民研究院为主的云计算中心、兴县轻铝合金为主的铝工业发展成为省委省政府关心支持的重点，全市开放的政策进一步完善，开放的格局初步形成。一年来，我们积极回应人民群众的热切期盼，困难面前不退缩、问题面前不回避，稳妥推进涉及7861户拆迁群众的新区安置区建设、以及涉及面更广的金融风险化解等工作，在解决遗留问题和难点问题中彰显了担当意识、彰显了奉献精神，广大干部干事创业的氛围逐渐形成。一年来，我们解放思想，改革创新，积极运用基金、 PPP 等现代金融工具，争取国开行、农发行贷款102亿元，有力支撑了脱贫攻坚、棚户区改造和城镇基础设施等重点项目建设；积极运用微信等现代信息化工具，推行重大项目“24小时直通车”制度，交流了工作经验，提高了工作效率。金融工具的运用、管理手段的创新，进一步推动了广大干部群众思想的解放，全市改革创新的气氛日益浓厚。一年来，我们坚持以脱贫攻坚统揽经济社会发展全局，特别是去年10月份以来，出台了一系列推进办法，认识更加到位、责任更加明确、措施更加精准，脱贫攻坚的被动局面得到了扭转，以脱贫攻坚统揽经济社会发展全局的认识，已经化作了全市广大干部群众的具体行动。以省第十一次党代会为标志，吕梁同全省一样翻开了新的一页，发展已经成为主旋律、最强音，吕梁已经全面开启不断塑造美好形象、逐步实现振兴崛起的新征程！

　　成绩来之不易，奋斗蕴含艰辛。这是省委、省政府亲切关怀、坚强领导的结果，是市委统揽全局、正确领导的结果，是市人大有效监督、市政协大力支持的结果，是全市广大干部群众顽强拼搏、狠抓落实的结果。在此，我代表市人民政府，向各位人大代表、政协委员、各民主党派、人民团体和各界人士，向驻吕解放军、武警官兵和公安干警，向全市人民及所有关心支持吕梁发展的朋友们，表示衷心的感谢和崇高的敬意！

　　各位代表，看到成绩的同时，我们也要直面问题。全市结构性矛盾仍然没有根本改变，煤焦冶电等传统产业仍然占到工业经济的70%，新兴产业尚未形成规模；经济企稳向好的基础还不牢固，经济增长仍然缺乏大项目支撑，新要素新动能和人才要素明显不足；企业负债率偏高、区域性金融风险仍然存在；城市供热供气等公共服务功能配套滞后，城市扩容压力巨大；贫困农民增收不稳定，脱贫摘帽奔小康任重道远；教育、医疗等民生事业欠账较多，补短板任务艰巨；生态环境脆弱，安全生产隐患较多，维稳压力较大；一些干部不敢为、不会为、不善为，一些部门和地方服务意识不强、行政效率不高、工作落实不力，等等。对此，我们将高度重视，认真解决，决不辜负人民的重托！

　　二、2017年工作安排

　　2017年是脱贫攻坚的关键之年，也是供给侧结构性改革和转型综改的深化之年。政府工作的总体要求是：深入学习贯彻习近平总书记系列重要讲话精神和治国理政新理念新思想新战略，统筹推进“五位一体”总体布局，协调推进“四个全面”战略布局，按照省委“一个指引、两手硬”重大思路和要求，认真落实骆惠宁书记对吕梁提出的扎实组织扶贫攻坚、扎实推动转型发展、扎实坚持全面从严治党“三件大事”要求，突出“六个坚持”，实现“六新目标”，坚持稳中求进工作总基调，全面做好稳增长、促改革、调结构、惠民生、防风险各项工作，全力促进经济稳步向好和社会和谐稳定，为决胜全面小康、塑造美好形象奠定更加坚实的基础，以优异成绩迎接党的十九大胜利召开。

　　主要预期指标是：地区生产总值增长5.5%，一般公共预算收入增长4%，规模以上工业增加值增长5%，全社会固定资产投资增长目标根据新的统计口径研究设置，社会消费品零售总额增长7%，城镇居民人均可支配收入、农村居民人均可支配收入分别增长6%和6%以上。城镇失业登记率控制在4%以内，城镇新增就业4万人。粮食总产量9.5亿公斤。

　　约束性指标是：万元 GDP 综合能耗下降率、万元 GDP 二氧化碳排放量、万元 GDP 用水量降幅等减排指标，完成省定任务。环境空气质量综合指数继续保持全省前列。2017年总体要求和主要指标设定，贯彻了省第十一次党代会精神和骆惠宁书记对吕梁提出的“三件大事”要求，坚持了稳中求进工作总基调。“稳”就是要确保经济基本面稳定、社会大局稳定、安全生产形势稳定和社会舆情稳定。“进”就是要撸起袖子大干快干加油干，力争发展速度再快一点， GDP、 规模以上工业增加值、一般公共预算收入等主要指标实现在全省“保中游、争上游”目标。“进”就是要以提高质量和效益为导向，加大改革开放力度，加快转型发展步伐，力争发展质量再高一点，努力在重点工作的推进上在全省乃至全国取得一定的影响，在重大民生问题的解决上取得让老百姓感受到、看得见的成效。

　　围绕以上目标，重点抓好以下工作：（一）坚持以脱贫攻坚统揽经济社会发展全局，坚决打赢脱贫攻坚战。

　　按照习近平总书记“六个精准”和骆惠宁书记“五个要”的要求，围绕实现“两不愁、三保障”目标，认真落实中央1号文件精神，把扶贫开发、现代农业发展、美丽乡村建设有机结合起来，扎实推进“春季行动”，认真实施“八个一批”，确保年内文水、汾阳、孝义3个非贫困县整体脱贫，方山、柳林摘帽，全市418个贫困村退出，14万贫困人口脱贫。

　　坚决啃下易地扶贫搬迁“硬骨头”。按照“五年任务三年完”“两年算总账”总要求，坚持安居与乐业并重，搬迁与脱贫同步，年内搬迁4.1万人，切实解决“一方水土养不活一方人”的问题。坚持易地搬迁、土地利用、城镇建设和产业发展多规合一，科学整合搬迁建房、基础设施配套等政策资金，积极落实搬迁地块，精准确定搬迁对象，全面加快搬迁进度。积极探索旧村复垦、沟域治理等土地利用有效路径，加大搬出村退耕还林、产业扶持力度，因地制宜规划好安置区后续产业发展，稳定增加搬迁群众经营性收入和资产性收入，确保搬得出、稳得住、能致富。

　　扎实推进特色产业扶贫。以创建全国农业标准化示范市和全国农业可持续发展示范市为抓手，全面落实各项强农惠农补贴政策，推进“一村一品一主体”产业扶贫行动，不断提高精准度。以功能农业为引领，主打“特色”“优质”两张牌，年内建设有机绿色无公害杂粮基地66万亩、马铃薯60万亩，发展食用菌4000万棒、中药材2.5万亩、牛羊养殖户6000个，实施退耕还草与粮改饲7.8万亩，重点打造兴县绿色谷子、临县肾型大豆、岚县马铃薯、方山中药材、交口食用菌、孝义设施蔬菜、汾阳核桃、文水交城养殖等特色产业基地县，努力把地方土特产和小品种做成带动农民增收的大产业。大力培育家庭农场、专业合作社等新型经营主体，扶持发展农村电商，开展农产品进北京、进省城、进高校活动，加快吕梁农产品“走出去”步伐。

　　打赢增绿与增收两场战役。我市沿黄生态环境差、水土流失严重、贫困人口集中。我们要紧紧抓住国家支持沿黄生态建设最给力的政策机遇，广泛推广政府购买式、合作式等多种造林模式，在生态治理战场上把吕梁打造成打赢增绿与增收两个战役的全国示范区。年内完成营造林105.78万亩，完成新一轮退耕还林81万亩，完成城区可视山体绿化5.3万亩，同时加大通道绿化、机场绿化、村庄绿化力度，让绿色成为吕梁的底色，让造林成为吕梁的时尚。有造林任务的贫困乡村要积极成立造林合作社，建档立卡贫困户占到合作社成员60%以上，让更多有劳动能力的贫困人口，在参与造林绿化、森林管护中获取更多的劳务收入，让吕梁山变成绿水青山、变成贫困群众增收的金山银山。

　　全力推进“三个一”扶贫计划。深入实施光伏扶贫，在10个贫困县（区）铺开惠及285个村、总规模189兆瓦的光伏电站，年内建成并网发电；积极探索收益分配办法，保障无劳动能力的深度贫困户优先受益、村集体收益稳定增加。扎实做好护工护理培训，加大宣传发动，年内培训1.25万人、就业1万人。继续推进经济林提质增效，实施红枣、核桃经济林提质增效20万亩、品种改良6万亩，发展沙棘、油用牡丹和构树8万亩，让荒山荒坡上的光伏扶贫、吕梁山的红枣核桃和吕梁山护工品牌一样，叫响山西、叫响全国，切实增加贫困人口经营性收入。

　　继续深化精准帮扶工作。大力加强“三基”建设，加大经费投入和教育培训力度，落实好“三支力量”帮扶工作责任制，实施“六个一”计划，提升基层组织和帮扶力量精准扶贫、精准脱贫的能力。扎实开展党员干部结对帮扶、技能培训、文明家庭创建等活动，提升贫困群众自身的脱贫能力，激发脱贫的积极性和主动性。完善县际结对帮扶机制，重点帮扶产业发展、学校医院等公共服务设施建设。积极引导各类企业参与贫困村产业开发，大力开展“民营企业吸纳贫困人口就业行动”“百企百村结对帮扶”等活动，积极推广大象“1+1+1+1”、泰化“三转三化”、厚通“公司+基地+合作社+农户”等产业扶贫模式。大张旗鼓地宣传表彰一批在脱贫攻坚战场上作出积极贡献的一线干部、帮扶企业和爱心人士，努力营造全社会关注扶贫、支持扶贫、参与扶贫的良好氛围。

　　着力深化脱贫攻坚系列改革。扎实推进挂图作战和痕迹管理，建立市级信息管理大数据平台，坚持在动态中识真贫、扶真贫、真脱贫。扎实推进贫困线和低保线“两线合一”兜底脱贫，让无劳动能力的贫困人口通过兜底实现稳定脱贫。改革财政专项扶贫资金使用管理方式，加大资金整合使用力度，提高财政资金的使用效率。推进政府 +银 行+保险+实施主体+贫困户“五位一体”的金融扶贫模式，增加产业扶贫贷款和扶贫小额信贷投放。总结推广石楼沟域经济、方山桥沟模式等资产性收益分配模式，拓宽贫困户增收渠道，努力推动户户有增收项目、人人有脱贫门路。

　　各位代表，脱贫攻坚是第一个百年奋斗目标最艰巨的任务。我们要把责任牢牢地扛在肩上，把任务紧紧地抓在手上，靠一项一项工作的扎实推进、靠一项一项工作走在全省前列，来确保小康路上不落下一个贫困村、不落下一个贫困户！

　　（二）坚持加快转型升级，实施产业发展战略。扎实推动转型发展是骆书记对吕梁提出的“三件大事”之一，是吕梁经济振兴的必由之路和治本良方。我们要以企业为主体、产业为牵引，以一个产业一个领导组、一个行业协会、一支基金、一个发展规划“四个一机制”为抓手，依托高科技、大资本和优秀人才，积极探索转型升级路径，不断提高吕梁经济发展的质量和水平。

　　大力培育新兴潜力产业。大数据产业，加快“天河二号”超算中心和“吕梁一号”微纳卫星数据产业化、市场化应用。年底前建成投运华为大数据中心。加快离石与北京协同创新研究院国家数据产业园项目建设，推进支付宝+吕梁合作项目初步建成；推动华唐集团大数据服务外包产业基地项目落地。积极与保利通信、世纪互联等一流大数据领军企业合作，努力将吕梁打造成承接省级和跨区业务的大数据管理节点、打造成信息高速核心汇聚节点。新材料产业，重点推进山西中磁尚善科技公司软磁电感、交口兴华290万吨铝基新材料、孝义信发20万吨氯化石蜡等项目建设。光能产业，充分发挥文水晋能清洁能源和离石晶旭新能源的人才、技术优势，以企招商，重点推动晋能1100兆瓦太阳能电池组件、国利天能600兆瓦太阳能反射镜等项目投产。机械制造业，重点推进交城泰集重工2000辆专用车、文水华一重工重型设备制造和大型锻件及机加工技改等项目建设。

　　发展壮大新的优势产业。铝工业，以电力体制改革为突破口，与全国排名前十的铝深加工企业通过项目捆绑、电力参股等合作模式，推进煤电铝材一体化发展，打造兴县、孝义、交口、中阳和柳林铝工业基地。支持中铝和兴县3月份组织铝材加工招商引资活动，实现9月份中铝华润50万吨轻合金项目建成投产的同时，上马一批铝型材系列深加工企业的目标。电力行业，加快孝义晋能发电项目建设进度，推动离石晋能大土河、临县京能2个低热值煤发电项目投产。白酒产业，支持汾酒集团做大做强，支持中汾10万吨白酒项目早日达产，支持汾阳建立国家级白酒检验检测中心和白酒交易中心，鼓励汾阳、文水、方山中小微白酒企业与汾酒集团合作，抱团生产、抱团经营，打造全国最大的清香型白酒生产基地。

　　改造提升传统产业。焦化产业，以孝义和交城经济开发区为主体，积极引导煤焦企业向煤化工产品高端化方向发展，重点推进孝义金岩250万吨焦化、金达150万吨焦化、金州5万吨煤系针状焦项目尽快投产；推进孝义鹏飞40万吨费托合成蜡、金岩45万吨甲醇联产3亿立方米 LNG、 金达2×5亿立方米 LNG、 金晖18万吨合成氨及配套干熄焦发电等项目开工建设，加快交城宏特煤系碳纤维、石墨烯电极、锂电池项目复产。钢铁产业，重点推进中钢一期50万吨线材拔丝项目建设，推进文水海威1800立方米高炉和120吨转炉全面复产。

　　把文化旅游业培育成战略性支柱产业。充分利用我市丰富的自然生态、历史文化、红色文化、廉政文化等资源，将资源优势转化为市场优势、竞争优势，加快形成新的增长动能。改造提升200公里沿黄旅游公路，建设以碛口古镇为重点的百里黄河旅游带，打造“黄河·碛口”旅游目的地。支持交城、岚县争创国家级全域旅游示范县。推进交城、方山、孝义、离石创建国家5A级景区工作，重点打造北武当山、庞泉沟、苍儿会、西华镇等特色景区景点。进一步完善旅游景区标识牌、厕所、停车场和商场、酒店等基础设施。推动141公里农村旅游公路建设上等级。改造提升离石—碛口旅游公路。推动旅游业与名人、名地相结合，加快建设于成龙廉政文化园，打造中汾酒城旅游区，丰富旅游文化内涵，打响吕梁旅游文化品牌。以建立一支旅游产业基金，引进一批战略投资者，实施一批旅游基础设施项目，举办一场旅游专场招商推介会，开展一次旅游形象宣传活动，支持县市举办“枣儿红了”“土豆花开了”和晋陕蒙民歌大赛等一系列活动的“六个一”旅游行动，助推文化旅游业快速发展。

　　推动现代服务业发展。我市现代服务业发展相对滞后，第三产业增加值仅占 GDP 的三分之一，对经济增长贡献率远低于全省、全国。物流、金融、电子商务等产业，是我市转型升级的重要方面，我们要依托中南铁路大动脉优势，加快沿线集运站建设。加快离石、汾阳、兴县物流信息服务平台和货物配载中心建设，在新区启动铁路货运物流区建设。深化金融开放与合作，积极发展互联网金融，探索股权众筹、债权众筹等新模式，提高企业直接融资能力，帮助解决困难企业融资问题。推广汾阳九都村镇银行做大做强的经验，推动村镇银行、小额贷款公司发展。启动离石、柳林、兴县信用社改制农商银行。积极推进电子商务进农村进社区，推进兴县、临县与阿里巴巴农村淘宝深入合作，推动岚县阿里巴巴农村淘宝项目尽快落地。大力推动实体零售创新转型，促进线上线下融合。围绕文化、体育、健康、养老等新消费热点，挖掘潜力，培育新的增长极。

　　各位代表，传统产业与新兴产业之间并没有万丈鸿沟，只要产业插上科技的翅膀、企业用了现代化的管理模式、企业家有了创新的精神，传统产业就一定能延伸催生出新兴产业。只要我们主动转型、创新转型、深度转型、全面转型，全市经济就一定能走出一条转型升级、集群发展的康庄大道！

　　（三）坚持供给侧结构改革与转型综改相结合，推进事关发展的关键性改革。

　　深化“供改”、加大“综改”既是经济工作的主线，又是改革创新的总抓手。向改革要红利，用改革促发展，我们必须大胆探索、勇于实践。

　　大力推进“三去一降一补”。做到“加减乘除”并用，不断提高供给体系的质量和效益。坚持去产能与推动优质产能提升相结合，引导煤炭企业退出产能60万吨，加快推进安全高效矿井建设，年内新增安全高效矿井10座，安全高效矿井数达到42座，占比达到70%。依法有序引导资不抵债、扭亏无望的水泥等行业“僵尸企业”关停退出，防止已经化解的过剩产能死灰复燃。坚持去库存与促进房地产平稳健康发展相结合，落实好出台的房地产去库存、棚户区改造货币化安置等政策，力争货币化安置比例达到60%以上。新开工棚改房1.18万套，重点推进市区凤山、世纪广场等棚户区示范区改造，加快全市棚户区改造步伐，确保三年棚改计划全部完成。坚持去杠杆与防范化解金融风险相结合，依法合规加快推进联盛集团金融风险化解工作，妥善处置临县白家峁矿权纠纷为重点的中诚信托风险。鼓励金融机构降低贷款利率、优化融资结构、延长贷款期限，降低企业杠杆率。坚持降成本与改善企业管理相结合，继续推进电力直接交易，以大用户直购电等模式降低耗能企业用电成本,力争完成交易电量28亿度。进一步优化企业发展环境，强化企业管理，降低企业税费负担和融资、财务、物流、制度性交易成本，切实提高效益。坚持补发展短板和补制度短板相结合，完善补短板项目储备和推进机制，重点在增强公共服务能力、加强基础设施薄弱环节、强化民生保障和生态建设等领域加大力度，切实提高补短板的精准性和有效性。

　　抓好关键环节和重点领域改革。深化国企国资改革，坚持市场化、法制化取向，把国企改革与国资管理改革、企业干部人事和人才制度改革统筹起来，重点推进离柳焦煤集团、吕梁中药厂等重点国有企业改革，妥善解决改制企业遗留问题。推进民企股份制改革，借鉴孝义市引导企业通过债转股、引进上市公司参与股权重组实现转型发展的经验，重点推进10户民营企业采取多种方式，与高科技和上市公司合作，试点股份制改造，着力提升企业现代管理水平。深化农村产权制度改革，基本完成农村土地承包经营权确权登记颁证任务。大力推进以光伏扶贫为重点的资产收益扶贫试点工作，探索“资产资源变股权、资金变股金、农民变股民、收益有分红”等增加农民财产性收入的新渠道。加快电力体制改革，以吕梁铝循环产业园区为试点，积极探索局域电网运营新模式，努力完成好试点任务，力争走在全省前列。加快旅游管理体制改革，推进碛口、北武当山、卦山和苍儿会等旅游景区管理体制机制改革创新，进一步理顺管理体制、经营体制和各种利益关系。引进一批战略合作者参股投资或参与管理，实施旅游景区景点专业化、公司化、市场化运营。加快投融资体制改革。积极运用产业发展基金，发行企业债券，撬动金融资本和社会资本，支持实体经济转型发展。充分发挥水务、国投、交通投资公司作用，加快推进城建、交通、水利等基础设施建设。积极推广“助保贷”等融资模式，通过政府增信、建立风险补偿金等融资方式，发挥财政资金杠杆作用，有效缓解中小企业融资难、融资贵问题。加快行政审批制度改革。深入推进“放管服效”改革，全面推行一个窗口、限时、网上办理，提高行政效能。大力推进“互联网+政务服务”，加快全市政务服务“一张网”建设步伐，着力提升政务服务便捷化和便民化水平。扎实推进城市管理执法体制改革，合理划分市区两级、部门和新区、政府和开发区及企业的城市管理事权、公共设施建设权、经营权，厘清责任，不断提升城市管理和服务水平。启动市辖区行政区划调整，稳妥推进县改区，逐步解决“一市一区”问题。

　　（四）坚持以开发区建设为平台，提高开放型经济发展水平。

　　加快开发区建设，是先进地区快速发展的成功经验。我们要以开放的胸怀，对标一流，先行先试，把开发区建设成对外开放的桥头堡、新兴产业的聚集区、民营经济发展的示范区、科技和人才的落脚地，努力提高开放型经济发展水平。

　　加快开发区改革创新发展。省委省政府十分重视开发区建设，明确提出“三化三制”改革，要求加快整合改制扩区调规步伐。我们要按照要求科学修编全市开发区总体发展规划，重点推进吕梁高新技术开发区、孝义经济开发区和汾阳、交城、文水经济开发区建设；加快兴县、岚县等8县“五规合一”，力争“十三五”末，实现市有国家级经济开发区、县有省级开发区目标。孝义经济开发区先行示范，6月底前完成“三化三制”改革，理顺开发区管理体制、运行机制和各类利益关系，建立责任明确、分工协作、运作高效的社会事务管理运行模式。坚持山上县与山下县统筹、一二三次产业统筹、传统产业与新兴产业统筹，依托比较优势，突出产业特色，实施错位发展，吕梁高新技术开发区主要布局大数据产业，孝义开发区主要布局煤电铝材产业，汾阳杏花村开发区主要布局白酒产业，交城开发区主要布局煤化工和大数据产业，文水开发区主要布局新能源和装备制造业，兴县开发区主要布局煤电铝材深加工产业，岚县开发区主要布局冶金和制造业。方山、临县主要推进全域旅游文化建设。柳林、交口、中阳、石楼等功能区、限制区，当前围绕现有主导产业，抓好现有产业基地建设。

　　推进科技和人才支撑。“山不辞土，故能成其高；海不辞水，故能成其大”。经济结构调整越是深入，科技和人才巨大作用越是凸显。要坚持需求导向和产业化方向，每个开发区建设一个省级科技企业孵化器和省级工程技术研究中心。积极帮助企业争取省级技术改造资金，配套市县技术改造资金，支持和引导企业开展技术改造。全力推进“百千万”人才工程和“百校千人”计划，深化与国防科技大学、北京航空航天大学、北京理工大学等高等院校的交流合作，积极引进企业和高等院校、科研单位、企业家和教授共建院士专家工作站、技术研发中心、科技服务站；组织好“院士吕梁行”活动，积极推动院士专家及其创新团队在吕梁开展科研攻关、技术诊断、人才培养、项目对接等工作。加强与省科协的战略合作，推进在各乡镇、重点村、重点企业、重点中小学以及公共场所建设科普e站，力争2017年实现全覆盖，让科技的光芒照耀在各个领域、影响到各个层面。

　　推动民营经济发展壮大。多年来，我市民营经济在 GDP 贡献率、提供就业岗位、上缴税收等方面发挥了重要支撑作用，为推动吕梁经济发展作出了突出贡献。我们要认真落实习近平总书记构建“亲”“清”新型政商关系要求，毫不动摇地支持民营经济发展，理直气壮地支持民营企业家干事创业，坦荡真诚地同民营企业接触交往，让全社会感受到市委、市政府支持民营经济发展的坚决态度，形成关心和支持民营企业创新发展的良好氛围。积极与北京航空航天大学等院校合作，实施民营企业转型升级“三个一”培训工程，组织100名重点企业负责人参加国内与国外的高端培训、举办1000名企业管理人才培训、开展10000名企业技术骨干业务培训，引导民营企业家转变思想观念、思维方式和发展理念，重塑吕梁民营企业家新形象。坚持企业家市长联络员制度，加强与民营企业的联系，协调解决民营企业发展中的突出问题；举办民营企业与科技、资本融合发展论坛，推动民营经济再创辉煌。大力支持个转企、小升规、规改股、股上市，年内新创办小微企业3000户以上，新培育小升规企业30个以上，力争3户中小企业登陆“新三板”，对上市企业在省政府奖励50万元的基础上，市政府再奖励20万元。

　　精准招商引资。主动融入全省“东融南承西联北拓”战略，积极参与京津冀一体化、晋陕蒙沿黄经济圈，差异化承接长三角等发达地区加工贸易产业梯度转移，在产业发展、基础设施等方面开展合作。围绕产业链条、产业集群、产业目录，精心策划包装一批重点项目，积极探索在江浙沪等发达地区建立“异地孵化器”，实施专业招商、以商招商、委托招商，不拘形式引商引智。加大有效投资力度，真正形成大项目顶天立地、小项目铺天盖地，助推区域经济发展和脱贫攻坚。继续引深“迎老乡、邀客商、兴吕梁”活动，支持离石举办大数据产业发展论坛、汾酒集团和汾阳举办清香型白酒博览会、汾阳举办全国第一届作家节、兴县举办铝深加工企业招商引资会、在太原北京举办系列农副产品进城进校展销等活动，让会展、论坛和招商成为吕梁开放的舞台、创新的平台。

　　（五）坚持不懈推进基础设施建设，加快城乡一体化发展。

　　积极顺应人民群众对城乡发展的新期待，以“五城联创”为引领，以“一城十镇百村”建设为重点，着力提升城镇规划、建设、管理水平，努力建设和谐宜居、富有活力的美丽家园。

　　重点推进“一城十镇百村”建设。推动市区建设提档升级，以完善城市功能、提升城市品位为目标，加快智慧城市、海绵城市建设步伐，推进市政道路、地下综合管廊、集贸市场、公共文化、公共服务、小广场和小公园等设施建设，加大绿化、亮化、美化力度，打造显山露水、增绿添路、宜居宜业、功能完善的新兴城市。主城区启动凤山公园提质改造、滨河东路南延等十大工程建设，新区启动吕梁广场、两带三湖北延工程、北川河生态治理等十大工程建设。推进“十镇百村”建设。科学统筹城乡发展，重点打造产业特色鲜明、环境和谐宜居、彰显传统文化、设施便捷完善的10个特色小镇。加快推动方山大武、柳林李家湾等重点城镇发展，推进孝义、交城两个国家新型城镇化试点建设。精心培育以“环境整治示范村”、“美丽宜居示范村”为内容的100个示范村庄，争取建设“一点”、靓丽“一片”。

　　全面开展“五城联创”活动。坚持高标准、严要求，在全市大力开展卫生城市、园林城市、环保模范城市、文明城市和优秀旅游城市“五城联创”活动，优化城市环境，提升城市形象，增强城市综合竞争力。岚县巩固国家级卫生县城成果，孝义通过国家级卫生城市复审，市区和汾阳、交城启动创建国家级卫生城市（县城），石楼启动创建省级卫生县城，其他县结合实际确立创建目标，启动创建工作。继续深入开展城乡环境卫生集中整治，加快城市污水处理、生活垃圾处理、集中供热供气等公共服务设施建设，大力推进城市公厕、街头游园、森林公园建设，提高公共服务水平。

　　加快城镇基础设施建设。交通方面，以铁路、高速、国省干线、旅游公路建设为重点，形成开放大通道、旅游大走廊。全力做好北京—延安高铁吕梁段项目前期工作，太兴铁路年内开通客运，吕梁至太原城际列车上半年加开提速。全面启动93.8公里静兴高速、96公里祁离高速公路建设，加快推进103.7公里汾石高速前期工作；开工建设60公里国道209市区段、18.2公里国道307柳林城区段和72.2公里国道307线交城—汾阳段改线工程；实施500公里农村公路改造、600公里农村公路生命安全防护工程；开工建设新区客运枢纽下安汽车站。水利方面，加快推进中部引黄县域小水网10处分水口、17座调蓄水库的前期工作；完成龙门供水主体工程建设，做好峪口沟水库前期工作，争取尽早审批立项。气化方面，积极推动国新燃气公司在离石枣林、临县林家坪、兴县蔡家崖、交口温泉等乡村试点供气，进一步提高乡村气化率。城市建设是城镇化发展的重点任务，我市城市建设起步较晚，基础设施建设比较落后，我们要以基础设施建设为重点，以宜居城乡为目标，努力改善人居环境，让人民群众生活的更方便、更舒心、更美好。

　　（六）坚持保障和改善民生，加快社会事业发展。

　　加快教育事业发展。积极回应群众反响强烈的教育问题，以市区为重点，扎实推进义务教育均衡发展，通过新建一批、改扩建一批、置换一批、盘活一批、扩规一批、购买一批等办法，优化各类学校布局，提升办学质量，实现一年大起步、二年见成效、三年大提升。幼儿教育，下决心解决公办幼儿园数量不足的问题，市区筹建9所公办幼儿园，推动恒大华府等5个小区配套建设公办或普惠性幼儿园，提高公办幼儿园比例。中小学教育，重点解决区域布局不合理、师资力量不足和教育质量不高的问题，市区新建改扩建小学10所、初中4所，整体搬迁离石二中，建成投用离石盛地小学、西属巴中学、方山贺龙中学；启动新区吕梁一中建设，扩大离石江阴高中办学规模。兴县、临县、石楼通过国家义务教育均衡发展评估认定。加强与省教育厅合作，开展中小学顶岗支教；试点推进方山、孝义、离石中小学人事制度改革，加强校长、教师队伍管理，加大师资培训力度，培育优良师德师风，全面提升教育质量。中职教育，重点是找准定位，创新模式，整合发展，培养更多实用型、技能型人才。大学与大专教育，要支持吕梁学院建设省内一流高校，争取北京航空航天大学与吕梁职业技术学院合作办学。开工建设吕梁高等师范专科学校。在全社会进一步形成关心教育、支持教育、重视教育、投资教育、尊师重教的良好风尚。

　　推进健康吕梁建设。深入实施“人才强卫”战略，利用国家卫计委在我市扶贫的机会，建立与北京医疗专家的联系合作机制，依托延安大学培训乡村医生，提高医务人员素质能力和业务水平。加强和省内外先进医疗机构的合作交流，高度重视医护人员招聘、培训、激励等问题，补齐医疗卫生服务的人才短板。加快推进新区医疗卫生园区、市人民医院综合门诊楼等医疗卫生基础设施建设，满足人民群众医疗服务需求。推进市人民医院、汾阳医院与县级医院医联体建设。继续重视基层医疗服务机构建设，加强基础设施建设和社区、乡村医生队伍建设，让老百姓在家门口就能享受到优质的基本医疗服务。加强计划生育服务能力建设，完善全面两孩政策体系。加强健康知识宣传力度，推进全民健身和全民健康深度融合，倡导健康文明的生活方式。

　　着力加强社会保障。全面实施机关事业单位养老保险制度改革，实现城乡居民基本医保制度并轨。实施残疾人精准康复服务行动。在全市城乡低保标准每人每月提标20元的基础上，进一步提高农村低保保障标准，逐步实现兜底脱贫。健全完善特困人员救助供养制度，供养标准达到低保标准的1.3倍。完成市儿童福利院主体工程，开工建设市社会福利院，县级综合福利服务中心实现全覆盖。深入实施高校毕业生就业创业促进和就业援助专项行动，努力增加就业岗位和创业机会。扎实推进拖欠农民工工资专项整治行动，切实维护农民工权益。深入开展食品药品安全综合治理，切实保障人民群众舌尖上的安全。

　　繁荣发展文化事业。深入挖掘我市丰富的文化资源和人文积淀，增强文化软实力。加快市图书馆、群艺馆建设，完善市体育场维修改造工程。加快推动县级“三馆一院”和基层综合性文化服务中心标准化建设。实施“书香吕梁”工程，推出更多有地方特色的精品力作。广泛开展群众性文化体育活动，丰富群众精神文化生活。加强非物质文化遗产保护。开展“传承好家风、争创文明户”活动，推动移风易俗，树立文明乡风村风。

　　重视国防教育、人防和兵役工作。加强文物保护、抗震设防、民族宗教、档案方志工作。支持各民主党派和各人民团体工作。做好统计、老龄、对台、气象、外事侨务等工作。

　　各位代表，今年，我们将围绕群众关心关注的热点难点问题，在完成省政府“六件实事”的基础上，集中力量办好我市的“六件民生实事”。一是孝义、汾阳、文水、柳林、方山等5个县（市）农村低保保障标准高于扶贫标准，实现低保线与贫困线“两线合一”；二是落实贫困户大病补充、意外险等扶贫保险1800万元，确保48万贫困人口全覆盖；三是市区建设公共自行车站点100个，投放2000辆公共自行车；四是完成农村危房改造14383户；五是解决14万建档立卡贫困户的饮水安全问题；六是完成4.2万座农村无害化卫生厕所改造。对这些民生实事,我们要一件不漏地办实办好,让老百姓的日子越过越好、越过越幸福。

　　（七）坚持全面加强生态环保和安全稳定，进一步改善发展环境。

　　牢记以人民为中心的发展思想，坚持标本兼治、综合治理、系统建设，坚决守住不发生重大环境事件、重大安全生产事故、重大群体性事件、重大金融风险“四条底线”，让人民群众在良好的环境中生产生活。

　　扎实推进“铁腕治污”行动。深刻汲取去年各类污染环境事件教训，坚持问题导向，实施大气、水、土壤污染防治三大战役，下决心解决环境污染突出问题。实施“控煤、治污、管车、降尘”等举措，从严打击重污染行业土小企业死灰复燃，强化节能降耗，持续提升全市空气环境质量。建立重污染天气预警应急响应和联动机制，实施空气质量信息共享、污染排放联合控制、联动执法，有效防止雾霾天气。改善河流地表水环境质量，重点推进工业废水处理提质、畜禽养殖粪便尿液处理和城镇生活污水处理设施及收集管网建设，全面提升辖区主要河流水质。积极开展土壤污染防治和环保隐患排查，确保环境安全。

　　加大生态治理力度。严格执行《吕梁市柳林泉域水资源保护条例》，强化柳林泉域保护与管理。全面推行“河长制”。加大汾河流域生态治理力度，开展重点河道综合整治。完成水土流失初步治理面积32万亩。大力推进矿山生态环境治理和地质灾害防治，积极推进“身边增绿行动”，努力营造天蓝水净、地绿山青的发展环境。

　　狠抓安全生产。推进安全生产依法治理，严厉打击非法违法生产经营建设行为;突出抓好煤矿、非煤矿山、危险化学品、交通运输、建筑施工等重点行业领域专项整治；对重点行业、重点领域、重点环节和重大危险源采取有效的技术、工程和管理控制措施，加快构建风险等级管控、隐患排查治理两条防线；加大安全投入力度，全面推进安全标准化建设，加强安全应急管理，建立市级危化品应急救援基地和队伍，不断提升安全生产保障能力。

　　维护社会稳定大局。创新社会治理方式，健全立体化社会治安防控体系，继续深化“打黑除恶”专项行动，严密防范和依法严惩各类违法犯罪行为。落实重大决策社会稳定风险评估机制，防止侵害群众利益现象发生。充分发挥社区居委会在社会管理中的积极作用，努力实现网格管理全覆盖、自治服务无缝隙。依法强化网络管理，积极防范和处置突发性群体事件。

　　各位代表，抓好生态环保和安全稳定，是一项长久而艰巨的任务。我们要以铁的担当尽责、铁的手腕治患、铁的心肠问责、铁的办法治本，决不要带血的 GDP， 决不要污染环境破坏生态的 GDP， 努力创造有质量有效益真金白银的 GDP、 可持续发展的 GDP， 让蓝天白云、青山绿水成为吕梁的一张“新名片”！

　　三、全面加强政府自身建设

　　政府系统要以习近平总书记系列重要讲话精神为指导，认真落实骆惠宁书记扎实推进全面从严治党要求，把深入学习贯彻党的十八届六中全会精神作为当前的一项重要政治任务，进一步增强政治意识、大局意识、核心意识、看齐意识。扎实推进“两学一做”学习教育，严肃认真开展党内政治生活，把《准则》、《条例》各项规定把握精、把握准，以思想到位、行动对标带动政府职能转变，努力构建符合法治精神、充满生机活力、更加廉洁高效的人民满意政府。

　　建设法治政府。运用战略思维、创新思维、辩证思维和法治思维，提升治理能力。深化法治吕梁建设，实施普法规划。落实公众参与、专家论证、风险评估、合法性审查、法律顾问和集体讨论决定等行政决策制度。自觉接受人大及其常委会的法律监督、工作监督，自觉接受人民政协的民主监督，认真听取人大代表、政协委员及各民主党派、工商联、无党派人士、各人民团体的意见建议，办好人大代表意见建议和政协提案。

　　建设担当政府。认真落实省委激励干部担当作为干事创业办法和支持干部改革创新合理容错办法，坚持“三个区分开来”，鼓励敢作为、善作为，宽容合理失误，让作风正派、敢打硬仗、锐意进取的干部在改革和攻坚的战场上有更多的底气和自信。加强政府系统干部管理，健全政府目标绩效考核指标体系和考评机制，对勤勉工作、业绩突出的干部，要予以褒奖。坚决纠正不作为、乱作为，坚决克服懒政、怠政，坚决惩处失职、渎职。继承和弘扬“吕梁精神”“胡兰精神”，始终保持奋发有为的精神状态，以“马上就办”的行动干事，以“钉钉子”的韧劲干事，真正做出经得起实践、人民和历史检验的成绩。

　　建设服务政府。全面清理涉及群众办事的各种不必要证明，让企业和群众办事更加便捷。推广电子政务和网上办事，力争年底前基本服务事项80%以上实现网上办理。做好“双随机一公开”与社会信用体系建设衔接工作，建设全市统一的公共信用信息共享平台，建立健全守信联合激励失信联合惩戒及红黑名单公示制度。

　　建设务实政府。深入践行党的群众路线，把政策向民生聚焦、服务向民生覆盖、资源向民生倾斜，带着深厚感情和强烈责任为群众办实事、解难题。全面落实“13710”工作制度，继续推进重点工程“3个24小时”工作制度，运用微信等现代工具，推动各项工作任务及时高效落实。“脚上有泥，心中才有数”。干部只有深入基层、深入实际、深入群众，到实践中经过一番捶打磨练，才能站得稳、立得住。我们要高度重视干部队伍建设，提升善谋良策、善于统筹、善抓落实、善于突破的能力，打造一支敢担当、有作为、作风硬的干部队伍，一支能够深入基层调查研究、“解剖麻雀”破解难题、推动发展维护稳定的干部队伍，努力塑造政府系统干部队伍新形象。

　　建设廉洁政府。认真落实各级政府党组全面从严治党的主体责任，严格执行党内政治生活准则，深入推进政府系统党风廉政建设和反腐败斗争。严格执行中央“八项规定”精神，始终保持惩治腐败、狠刹“四风”高压态势。牢固树立“过紧日子”思想，严控“三公”经费支出，把有限的资金花好用好。严肃整治关键领域、关键科室、关键人员推诿扯皮、吃拿卡要等问题，重点整治好门好进、脸好看、话好听、但事依然难办等问题。抓好廉政教育和廉政文化建设，完善防腐制度体系，着力构建不敢腐、不能腐、不想腐的长效机制。

各位代表，“弄潮儿向涛头立，手把红旗旗不湿”。让我们紧密团结在以习近平同志为核心的党中央周围，在省委、省政府和市委的坚强领导下，凝心聚力、锐意进取，勤勉工作、求实高效，以实际行动和优异成绩迎接党的十九大胜利召开！
